# Minimum accelerometer wear-time for reliable estimates of physical activity and sedentary behaviour of people receiving haemodialysis

**DOI:** 10.1186/s12882-020-01877-8

**Published:** 2020-06-16

**Authors:** Sean Prescott, Jamie P. Traynor, Ilona Shilliday, Tobia Zanotto, Robert Rush, Thomas H. Mercer

**Affiliations:** 1 Centre for Health, Activity and Rehabilitation Research, Queen Margaret University, Edinburgh, Scotland EH21 6UU UK; 2 Queen Elizabeth University Hospital, Renal and Transplant Unit, Glasgow, UK; 3 Monklands Hospital, Renal unit, Airdrie, UK

**Keywords:** Accelerometer, Actigraph, ActivPAL, Reliability, Wear-time, Physical activity

## Abstract

**Background:**

Low levels of physical activity are implicated in low life expectancies of people receiving maintenance haemodialysis. Accelerometers are increasingly being used to quantify activity behaviours of this population but guidance to quality-assure such data is lacking. The objective of this study was to provide data processing and reduction recommendations to ensure accelerometer-derived outcomes are sufficiently reliable for interpretative analysis.

**Methods:**

Seventy people receiving maintenance haemodialysis (age 55.9 ± 15.7 years, 34% women, 23% diabetic) from a single outpatient renal unit volunteered for the study. Participants wore Actigraph GT3x and ActivPAL monitors during waking hours over seven days. Reliability of accelerometer output (normalised to wear-time) was assessed via intraclass correlation coefficient (ICC). The Spearman-Brown prophecy formula was subsequently applied to the ICCs to derive the minimum required accelerometer wear-time for each behavioural outcome.

**Results:**

Monitor wear compliance was greater on dialysis compared to non-dialysis days (90% v 77%). Participants were significantly more active on non-dialysis days compared to dialysis days but there were no significant differences in estimated behaviours between days within the same condition. Average measure ICCs for all accelerometer outcomes were high (range 0.76–0.96). Computations indicated that habitual physical activity and sedentary behaviour could be estimated with a minimum reliability level of 0.80 from one dialysis day and two non-dialysis days, and at least eight hours monitor wear per day. Applying this rubric allowed 90% of participant data to be retained for further analysis.

**Conclusions:**

Regardless of accelerometer, one dialysis and two non-dialysis days data with a minimum of eight hours wear each day should enable habitual activity of people receiving maintenance haemodialysis to be characterised with acceptable reliability. These recommendations reconcile the tension between wear-time criteria stringency and retention of an adequately representative sample.

## Background

In the last two decades, a great number of studies have consistently documented that low levels of physical activity (PA) are highly prevalent among people receiving maintenance haemodialysis (MHD) for established renal failure (ERF) [1–5]. Large-scale epidemiological studies suggest that 35 to 44% of this clinical population never engage in any kind of structured PA, with 38 to 42% of patients having severe limitations in performing moderate PA [2, 3]. Interestingly, even higher proportions of low PA seem to emerge from studies which employed more objective measurements, such as accelerometry, as about two thirds of people receiving MHD were categorised as either low active or sedentary [6, 7]. Importantly, sedentary behaviour in people with ERF is shaped not only by high comorbidity levels and poor physical function [8], but also by the fact that MHD entails prolonged periods of sedentary activity due to the imposed sitting time during dialysis. Many studies have shown that patients are less active during dialysis days compared to non-dialysis days [9, 10]. The clinical implications of low PA levels involve multiple domains of health. For instance, sedentary behaviour negatively affects global physical function due to the well-known adverse effects on bone and skeletal muscle function [8], but also cardiovascular and mental health [11, 12]. Overall, low PA has been linked to lower quality of life as well as to the development of adverse clinical outcomes such as hospitalisations, major cardiovascular events and mortality in people receiving MHD [2, 3, 12]. The World Health Organisation target of a 10% relative reduction in the prevalence of insufficient physical activity by 2025 to reduce premature mortality indicates PA may shortly be monitored alongside physiological health indices [13].

While patient-reported PA questionnaires are expedient, they have recognised limitations and accelerometers are thus increasingly being adopted to objectively estimate PA and sedentary behaviour. Importantly, accelerometer derived outcomes are predictive of mortality, physical function and associated with indices of health status in the MHD population [4, 6, 7, 14–16]. However, if accelerometer-based PA surveillance is to be routinely employed in health management and research, then stringent methodological criteria should govern its use. A seven-day monitor wear period is typically prescribed [6, 7, 17], but despite reminders and incentives, compliance is often variable [18] with daily wear-time also subject to variation within and between participants [19]. The importance of minimum accelerometer-wear recommendations for reliable estimation of habitual PA has been highlighted [20, 21]. Nonetheless, previous studies using accelerometers to characterise PA of people receiving MHD have lacked methodological uniformity. Number and type of days monitored are inconsistent, often with no stated rationale, while more than 80% of studies failed to define the number of wear hours required to constitute a valid day. Moreover, these studies are remarkable for the fact that no participants were excluded from analyses due to insufficient monitor wear. Thus, it would appear they have not explicitly at least, taken into account the effects of discretionary wear on accelerometer outcomes.

There is a trade-off between applying wear criteria that are stringent enough to ensure data consistency while at the same time facilitating retention of sample size that is sufficiently representative and adequate for interpretative analysis [19, 22, 23]. Guidance for accelerometer data reduction is available for asymptomatic adults [24–27], but no equivalent methodological recommendations exist for clinical populations like ERF, perhaps indicating low awareness of their importance. In light of evidence linking PA and sedentary time with health outcomes, there is a pressing need to establish accelerometer data reduction guidelines for reliable estimation of these behaviours in ERF. The objectives of this study were to determine the minimum accelerometer wear-time necessary for reliable characterisation of habitual activity, and the impact of wear criteria on sample retention. The aim was to offer methodological recommendations to underpin quality assurance of future accelerometer-based research in ERF.

## Methods

### Study design, setting and participants

This study conformed to the Declaration of Helsinki and was approved by the West of Scotland Research Ethics Service (REC reference number: 11/WS/0001). Seventy-two self-selected individuals (55.9 ± 15.7 years) undergoing maintenance HD therapy (thrice weekly) at Monklands Hospital outpatient HD unit volunteered to take part in this reliability study, which was conducted between November 2011 and August 2013. Male or female individuals aged 18 years or over and able to ambulate a minimum of 10 m (use of a walking aid was acceptable) were considered eligible, while people with dementia or severe cognitive impairment were excluded from participation. Participant incentives were not used for this research project. Written informed consent was obtained from each participant.

### Demographic and clinical characteristics

Participant demographics (age, gender, body mass index), and clinical characteristics (biochemistry values, diagnosis of diabetes, dialysis vintage and adequacy) were extracted from the patients’ electronic medical records. Haemoglobin (Hb) and albumin values were recorded from the closest routine monthly bloods to the PA assessment (within the nearest two-week period). Participants travelled to the dialysis unit either by ambulance transport provided by the hospital or by car.

### Objective measurement of physical activity

Physical activity was estimated via Actigraph GT3x (Actigraph Corp, Pensacola, Florida) and ActivPAL (PAL Technologies Ltd., Glasgow) accelerometers, which were positioned as per the manufacturers’ specifications on the non-dominant hip and thigh respectively. The Actigraph GT3x records body accelerations within 0.05–0.2 units of gravity in three individual axes (vertical, horizontal, perpendicular), with a sampling frequency of 30 Hz. The raw accelerometer data are then converted into activity counts, with higher counts resulting from greater or more frequent accelerations. Step-counts are calculated based on accelerometer data recorded on the vertical axis only and are accumulated on a per-epoch basis. The ActivPAL is a uniaxial accelerometer that records posture outputs and step-counts from thigh inclination, with a sampling frequency of 10 Hz. Time spent in different postures, transitions and step-counts are derived via algorithms embedded in the ActivPAL software. Both accelerometers were synchronised and initialised to collect data over 15 s epochs [28] using the proprietary software for each device installed on the same laptop. Participants were instructed to wear the accelerometers simultaneously during waking hours for 8 days and were provided with a wear log.

### Accelerometer data cleaning and categorisation

Participants were habituated to the accelerometers for a day, which was excluded from subsequent analysis. The monitoring period included three dialysis days, and four non-dialysis days. Actigraph files were scrutinised for spurious data (> 20,000 counts per minute (cpm)) and wear-time was determined using a filter of 150 consecutive zero-count minutes, with allowance for < 1 min of activity counts < 100 cpm [29]. Data were inspected to ensure dialysis sessions were not misclassified as non-wear time. Actigraph files with > 18 h/day wear-time suggested to be implausible [30] had logical waking hours triangulated via ActivPAL data and the monitor wear log. ActivPAL files were scrutinised for monitor malfunctions using the PAL Technologies software. Data were then exported to an Excel spreadsheet to enable accurate determination of monitor wear (time between first and last sit-to-stand transfers) and values for activity behaviours.

All three Actigraph GT3x axes were enabled for PA measurement. Sedentary time was determined via vertical axis data using a uniaxial cutpoint of < 100 cpm [31], with activity counts above this threshold categorised as total PA. Time spent in moderate to vigorous physical activity (MVPA) was categorised using a uniaxial cutpoint of ≥1952 cpm [32]. ActivPAL estimated sitting/lying was employed to indicate sedentary time in line with the current definition for this behaviour [33]. ActivPAL total PA was derived from time spent in standing activities, as energy expenditure of postural skeletal muscle is above the 1.5 metabolic equivalent of task (MET) threshold for sedentary behaviour [33].

### Data analysis

**Fig. 1:**
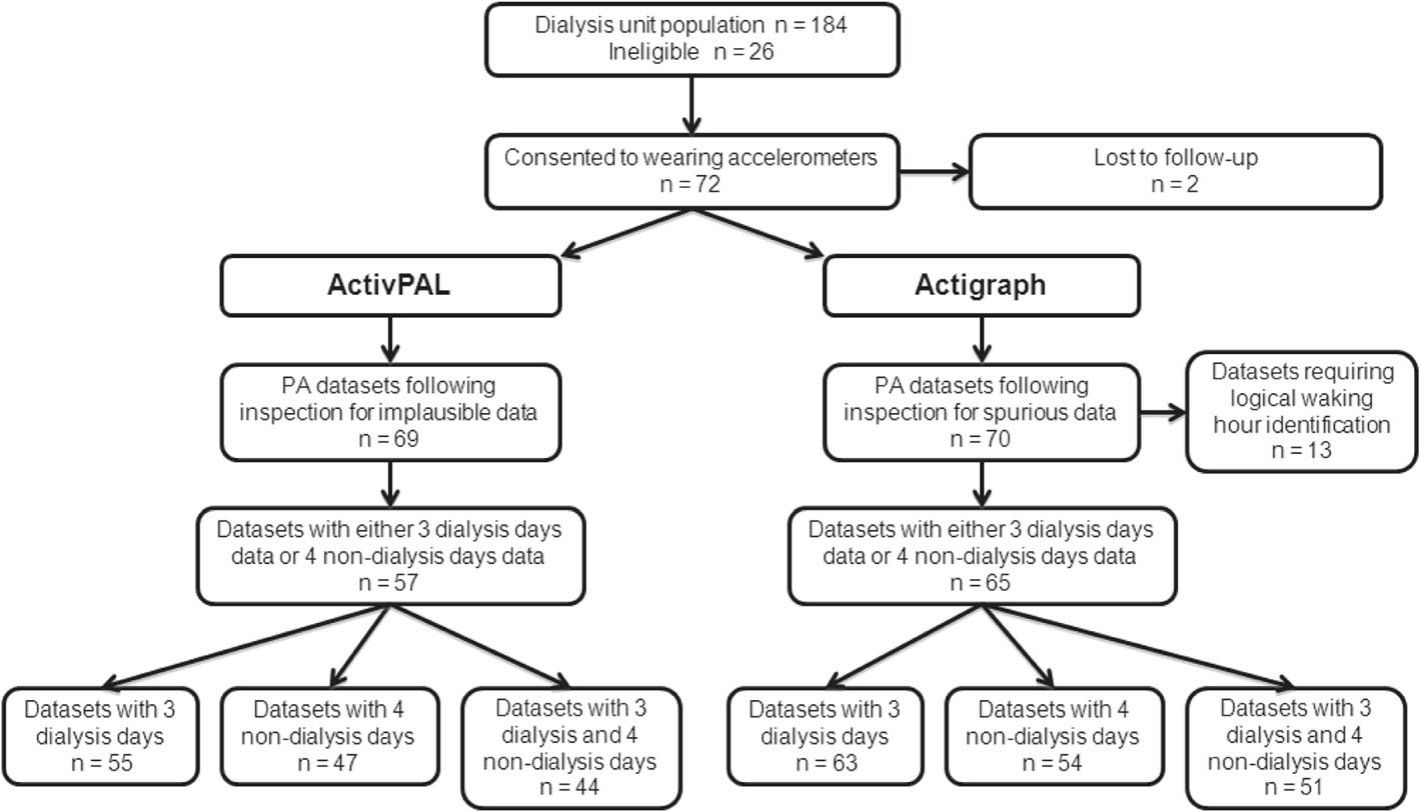
Flowchart of accelerometer data cleaning and participant inclusion for reliability analysis

Reported Actigraph outcomes include: sedentary time; total PA; MVPA; step-count; triaxial activity counts. ActivPAL outcomes include: sit/lie time; standing time; step-count; sit-to-stand transfers; energy expenditure. All outcomes were normalised to daily wear-time to adjust for variation in discretionary wear [19]. Missing accelerometer data due to loss to follow-up (*n* = 2, 2.8%) were handled with listwise deletion method in the analysis, while missing accelerometer data arising from incomplete compliance (range: *n* = 9, 12.5% to *n* = 28, 38.9%, as detailed in Fig. 1) were handled with pairwise deletion. Data are presented as mean and standard deviation (SD) or median and interquartile range (IQR) depending on the underlying distribution. Condition effect (i.e. dialysis versus non-dialysis days) on PA outcomes was tested via paired t-test or Wilcoxon signed rank test. Day effects within the same condition (i.e. dialysis days only) were tested via repeated measures ANOVA or Friedman’s test. Test significance level was set at *p* < .05. Variability of accelerometer outcomes for dialysis and non-dialysis days was assessed via intra-class correlation coefficient (ICC) employing a two-way random effects model (ICC _2,1_). Non-normally distributed variables were transformed for reliability calculations.

### Calculation of required accelerometer wear-time

Minimum required wear was computed using the Spearman-Brown prophecy formula [34], where ‘N’ equals the recommended number of wear days, ICC_D_ is the desired reliability level and ICC_SM_ is the single measure of reliability. A reliability level of not less than 0.80 is recommended for physiological data [35]. The influence of longer daily wear and different levels of desired reliability on required monitor wear was examined.

$$ \mathrm{N}=\left[{\mathrm{ICC}}_{\mathrm{D}}\left(1\hbox{-} {\mathrm{ICC}}_{\mathrm{D}}\right)\right]\ \left[\left(1\hbox{-} {\mathrm{ICC}}_{\mathrm{SM}}\right)\ {\mathrm{ICC}}_{\mathrm{SM}}\right] $$

If computed wear days exceeded a whole number, the required wear-time was rounded up to the next whole number to ensure a minimum reliability criterion was maintained. The impact of wear-time criteria on participant retention was also examined.

## Results

Figure 1 illustrates the data cleaning and participant inclusion process. Participant characteristics are presented in Table 1. Age ranged from 24 to 87 years with women making up one third of the sample, while diabetes prevalence was nearly one quarter. The mean prescribed time on dialysis per session was 4 h. Biomarkers of health and dialysis adequacy were within target ranges recommended by the UK Renal Association.

**Table 1:** Participant characteristics. [Mean ± SD, or Median (IQR)]

	Total sample	Males	Females
n	70	46	24
Diabetes	16 (23%)	11 (24%)	5 (21%)
Age (years)	55.9 ± 15.7	58.8 ± 16.2	50.2 ± 13.3
BMI (kg/m^2^)	28.6 ± 6.4	28.0 ± 4.7	29.7 ± 8.8
Albumin (g/L)	39.0 (36.0–42.0)	39.5 (36.0–42.0)	39.0 (36.0–41.0)
Hb (g/dL)	11.3 ± 1.0	11.4 ± 1.0	11.0 ± 0.9
Dialysis adequacy (URR %)	71.0 (66.0–75.0)	69.0 (65.8–73.0)	75.0 (70.1–79.0)
Dialysis vintage (months)	15.8 (6.8–32.0)	17.2 (8.2–31.1)	9.4 (5.4–34.2)

*BMI* Body mass index; *Hb* Haemoglobin; *URR* Urea reduction ratio

Actigraph and ActivPAL wear compliance for the seven-day monitoring period was 73 and 63% respectively, with higher compliance observed for all three dialysis days (90 and 79% respectively) compared to the four non-dialysis days (77 and 67% respectively). Averaged seven-day values for Actigraph and ActivPAL outcome variables are presented in Table 2. Participants were significantly more sedentary on dialysis days while total PA and other activity indices were significantly higher on non-dialysis days. No significant differences were observed for accelerometer output variables among days within the same condition (dialysis vs non-dialysis) (Additional file 1: Table S1).

**Table 2:** Accelerometer derived outcomes. [Mean ± SD or Median (IQR)]

	7 Days	Dialysis	Non-dialysis	Dialysis v Non-dialysis
**Actigraph GT3x**	(*n* = 51)	(*n* = 63)	(*n* = 54)	
Wear time (min)	798.8 ± 103.1	863.7 ± 124.9	750.2 ± 111.8	t (_50_) = 11.40 *p* < 0.001
Sedentary (%)	83.4 ± 8.9	86.4 ± 6.6	81.2 ± 11.0	t (_50_) = 6.021 p < 0.001
Total PA (%)	16.6 ± 8.9	13.6 ± 6.6	18.8 ± 11.0	t (_50_) = − 6.021 p < 0.001
MVPA (%)	0.9 (0.3, 2.3)	0.7 (0.3, 1.4)	0.9 (0.2, 3.0)	Z = − 3.21 *p* = 0.001
Steps/day	2303 (1048, 3876)	1935 (959, 2666)	2370 (1115, 4391)	Z = − 3.05 *p* = 0.002
Steps/min	2.8 (1.5, 5.0)	2.2 (1.4, 3.6)	3.3 (1.5, 5.4)	Z = − 4.04 p < 0.001
Triaxial cpm	303 (176, 397)	233(151, 327)	322 (203, 473)	Z = − 5.54 p < 0.001
**ActivPAL**	(*n* = 44)	(*n* = 55)	(*n* = 47)	
Wear (min)	801.7 ± 107.6	856.51 ± 134.8	745.05 ± 124.0	t (_43_) = 5.565 p < 0.001
Sit/Lie (%)	74.89 ± 12.07^†^	81.7 ± 9.5	70.0 ± 15.9	t (_43_) = 6.046 p < 0.001
Stand (%)	25.1 ± 12.1	18.3 ± 9.5	30.0 ± 15.9	t (_43_) = − 6.047 p < 0.001
Transitions/hour	2.6 ± 0.8	2.1 ± 0.7	2.9 ± 1.0	t (_43_) = − 7.568 p < 0.001
Steps/day	3242 (1909, 4615)	2189 (1493, 3577)	3594 (2055, 5854)	Z = − 3.769 p < 0.001
Steps/min	4.2 (2.5, 6.1)	2.76 (1.8, 3.7)	4.9 (2.6, 8.3)	Z = − 4.621 p < 0.001
EE (METs/min)	1.37 (1.33, 1.42)	1.34 (1.31, 1.36)	1.40 (1.33, 1.49)	Z = − 4.831 p < 0.001

*MVPA* Moderate to vigorous physical activity; *EE* Energy expenditure; *cpm* Counts per minute

Average measure ICCs for accelerometer outcomes ranged from 0.89 to 0.96 and 0.76 to 0.94 for Actigraph and ActivPAL respectively (Additional file 1: Tables S2-S5). Reliability of Actigraph outcomes remained consistently high with longer monitor wear-time, while ActivPAL reliability on non-dialysis days tended to decline as the number of participant datasets dropped below 45. Computed wear-times for Actigraph and ActivPAL outcomes are presented in Table 3. In general, the required number of dialysis-days for each outcome declines as the minimum amount of daily wear increases. Comparatively greater non-dialysis day wear is required.

**Table 3:** Computed minimum wear-time requirements for Actigraph and ActivPAL outcomes on dialysis days and non-dialysis days with a reliability level of 0.80

***Actigraph***										
	Sedentary %		Total PA %		MVPA%		Steps/min		Triaxial counts/min	
**Wear/day**	Dialysis	Non-dialysis	Dialysis	Non-dialysis	Dialysis	Non-dialysis	Dialysis	Non-dialysis	Dialysis	Non-dialysis
≥6 h	1.15	1.21	1.15	1.21	1.12	0.93	0.64	0.71	0.89	0.77
≥7 h	0.99	1.16	0.99	1.16	1.16	0.87	0.61	0.71	0.81	0.76
≥8 h	0.98	1.35	0.98	1.35	1.12	0.89	0.60	0.85	0.80	0.87
≥9 h	1.00	1.38	1.00	1.38	1.14	0.95	0.60	0.84	0.80	0.87
≥10 h	0.96	1.97	0.96	1.97	1.14	0.97	0.59	0.87	0.77	0.99
***ActivPAL***										
	**Sit/lie time %**		**Stand time %**		**Transfers/hour**		**Steps/min**		**Energy MET/min**	
**Wear/day**	Dialysis	Non-dialysis	Dialysis	Non-dialysis	Dialysis	Non-dialysis	Dialysis	Non-dialysis	Dialysis	Non-dialysis
≥6 h	0.80	1.93	0.80	1.93	2.76	2.06	1.15	1.19	0.83	0.83
≥7 h	0.79	1.93	0.79	1.93	2.90	2.06	1.12	1.19	0.82	0.82
≥8 h	0.79	2.27	0.79	2.27	2.90	2.03	1.12	1.35	0.82	0.82
≥9 h	0.83	2.54	0.83	2.54	3.03	2.46	1.25	1.41	0.85	0.85
≥10 h	0.72	1.99	0.72	1.99	2.88	3.14	1.21	1.50	0.70	0.70

Minimum required wear-time varied according to outcome. One dialysis day and more than one but less than two non-dialysis days were required for estimation of sedentary behaviour and total PA, with a reliability level of 0.80, from either accelerometer. A range of 1.12 to 1.16 dialysis and 0.87 to 0.97 non-dialysis days were necessary for Actigraph estimates of MVPA. Actigraph activity counts/minute and estimated steps/minute as well as ActivPAL estimated energy expenditure required 0.59 to 0.89 dialysis days and 0.70 to 0.99 non-dialysis days. A range of 1.12 to 1.5 days of each condition were required for ActivPAL step-counts while a total of 4.84 to 6.02 days were necessary for sit-to-stand transfers. Wear requirements were generally greater when data were not normalised to accelerometer wear-time, and more than doubled when reliability level was increased to 0.90 (Additional file 1: Tables S6-S13).

Table 4 illustrates participant compliance with accelerometer wear and retention according to one-hour increments of daily wear. Almost all participants returned Actigraph and ActivPAL data containing a minimum of one dialysis day and at least 84% wore the devices for two non-dialysis days. The proportion of participants retained declines as the required wear per day increases, and is governed largely by individuals returning sufficient non-dialysis day wear. The majority of participants (94%) were retained if the Spearman-Brown derived minimum-wear recommendations are applied.

**Table 4:** Number (and %) of participants retained according to minimum daily wear-time and required number of wear days

Wear time criteria	≥6 h		≥7 h		≥8 h		≥9 h		≥10 h	
	Actigraph	Activpal	Actigraph	Activpal	Actigraph	Activpal	Actigraph	Activpal	Actigraph	Activpal
Dialysis days ≥1	70 (100)	69 (98.6)	70 (100)	69 (98.6)	70 (100)	69 (98.6)	70 (100)	68 (97.1)	70 (100)	68 (97.1)
Non-dialysis days ≥2	66 (94.3)	66 (94.3)	65 (92.9)	66 (94.3)	63 (90)	64 (91.4)	63 (90)	62 (88.6)	61 (87.1)	59 (84.3)
Participants retained	66 (94.3)	66 (94.3)	65 (92.9)	66 (94.3)	63 (90)	64 (91.4)	63 (90)	61 (87.1)	61 (87.1)	59 (84.3)

## Discussion

This is the first study to provide comprehensive wear-time guidance for reliable estimation of activity of people receiving MHD. Wear-time requirements varied according to outcome, but overall, it appears that regardless of which accelerometer is employed, a minimum of any one dialysis and two non-dialysis days wear should provide reliable estimates of habitual PA and sedentary behaviour. The clinical characteristics of the study sample are similar to those of the wider Scottish MHD population [36]. The median age was younger than that of the whole dialysis unit (56 versus 64 years) and Scottish HD population [36] but similar to previous motion-sensor based PA studies involving people receiving MHD [6, 7, 16, 37].

Wear-time recommendations made here for total PA are in line with previous studies employing older uniaxial Actigraphs. Hart et al. [26] observed 3 days of Actigraph wear provided similar reliability for total PA estimates in a sample of 52 asymptomatic older adults (69.3 ± 7.4 years). Three to 4 days and four to 5 days of accelerometer wear has been recommended for healthy older adults [24] and young to middle-aged rural and urban adults [25] respectively. The discrepancy between these studies and the present study may be due in part to the former employing a higher total PA cutpoint (> 500 cpm). Higher wear requirements reported by Cook and Lambert [25] may also reflect greater variability of daily PA for younger adults. In contrast, people receiving MHD effectively have their activity clamped on dialysis days thereby limiting intra-day variability. In addition, reliability analyses in the cited studies were performed on minutes of total PA, and thus ignored the potential effects of inter- and intra-individual variation in discretionary wear on accelerometer output. This may also explain why the required wear-time for sedentary behaviour proposed here (3 days) is lower than the five to 7 days recommended in previous studies [24–26]. Computed wear-time requirements for minutes of sedentary time in the present study were similarly high at six to 7 days (Additional file 1: Tables S10-S13) due to lower reliability.

In our study, reliable Actigraph triaxial activity count output was obtained from just 2 days wear. Similarly, a study simulating triaxial accelerometry using three Caltrac devices produced comparable reliability (0.83) from 2 days data in a smaller sample of 30 asymptomatic young men [38]. Coleman and Epstein [39], reported three to 4 days of wear gave acceptable levels of generalisability for Tri-Trac-3D activity count output of young, low-active men. Overall, required wear-time for triaxial activity counts in the present study is consistent with two to 3 days reported for middle-aged to older adults using older uniaxial Actigraphs [24, 25, 40]. Three days of Actigraph wear were necessary for reliable estimation of MVPA, the type of activity associated with a health enhancing effect. This is in agreement with Matthews et al. [24] who recommended three to 4 days wear for healthy middle-aged adults. Four to 5 days was recommended for younger rural and urban adults by Cook and Lambert [25], a difference possibly mediated in part by MVPA often being planned and less variable for older adults [41], who made up a large proportion of the present sample.

To the best of our knowledge, only two reliability studies examined the minimum wear requirements for the ActivPAL monitor. The recommendations made by these studies, which were conducted in asymptomatic adolescent females [42] and adults [43], seem to partially contrast with our findings. Particularly, Dowd et al., [42] concluded that at least 12 days of accelerometer wear would be required to estimate sedentary behaviour in a female population, aged 13–18 years-old, with the same level of reliability used in the present study (ICC ≥ 0.8). On the other hand, Aguilar-Farias et al., [43] reported that, in order to reliably estimate PA and sedentary behaviour in a group of asymptomatic adults (mean age = 39.1 ± 12.4 years; ICC ≥ 0.8), at least 5 days of ActivPAL wear would be needed. The apparent discrepancies between these findings and the observations emerging from our study are almost certainly ascribable to the different populations examined. Specifically, we take the view that older age, comorbidities and lower levels of functional independence may be responsible for the lower inter- and intra-individual variability in PA behaviours exhibited by our study participants.

Step-counts are often used as a motivational outcome [19] and have previously been employed in ERF [37], however recommended wear-time has not previously been examined for accelerometers with this output. Minimum required accelerometer wear for steps/minute and steps/day was two to 4 days in our study, which is consistent with previous research evaluating pedometer reliability during free-living conditions. Any 2 days of pedometer wear were recommended by Rowe et al. [41] for senior adults (74.0 ± 9.5 years), while three to 4 days were required for asymptomatic middle-aged [44] and older adults [26] respectively. In contrast, Kang et al. [45] recommended up to 6 days, a disparity which may be due to much younger participant age (38 ± 9.9 years) and seasonality increasing step-count variability in their study.

Indices of PA were significantly higher on non-dialysis days compared to dialysis days, which is in agreement with earlier research [9, 10]. High average measure ICCs and no observed significant differences between days within the same condition (Additional file 1: Table S1) suggest the inclusion of ‘weekend’ wear is not compulsory. This finding is consistent with previous studies showing no additional improvement in accelerometer outcome reliability with weekend data inclusion for adults [46, 47].

Guidance regarding hours of accelerometer wear per day is crucial to determine which days may be included for subsequent analyses, as minimum daily wear-time impacts PA outcomes of participants and sample size retention [20, 23]. These data suggest that including days with as few as 6 h wear-time does not appear to adversely affect accelerometer outcome reliability. On balance however, a minimum standard of 8 h to ‘rule in’ a valid day is recommended as more appropriate due to the amount of time occupied by MHD and the variation in PA patterns around this event [15]. Previous studies have similarly concluded wear thresholds of six to 8 h per day facilitate acceptable reliability for PA estimation of children [48, 49]. This recommendation contrasts with the commonly employed standard of 10 h/day advocated by Troiano et al. [50]. However, the latter was adopted from research examining sample size retention according to different wear-time algorithms [20] and not reliability analyses.

Obviously, using the 10-h benchmark increases the ability to compare findings, but this wear criterion may not be feasible for everyone receiving MHD, a clinical population beset with high prevalence of multi-morbidity, condition-related symptoms, low PA and advanced average age. Moreover, the amount of daily wear-time appears to be less influential on PA outcomes in low-active populations, but significantly impacts sample size retention [51]. The rubric suggested here (one dialysis and two non-dialysis days, 8 hours/day wear) would allow 90% of our sample to be included for final analyses, and is comparable to the 93% retention rate reported by Chen et al. [27] who employed similar data reduction criteria for low-active individuals. Importantly, applying the ≥10 h/day criterion would reduce the present sample size by three to 7 % without an appreciable increase in reliability and possibly introduce a source of bias.

Minimum wear recommendations are necessary for PA data quality assurance so that the ability to detect relationships with other variables is not diminished and conclusions drawn are not limited [18]. Moreover, reliable characterisation of PA is crucial for comparison with previous research, as well as for monitoring purposes to stratify health risk, and detect PA behaviour change. Interestingly, Actigraph data revealed only 13% of our sample met recommended levels of MVPA for a health enhancing effect, however, 92% regularly achieved a threshold of 50 min of total daily PA, which is independently associated with improved survival in ERF [4]. It should also be acknowledged that, since low self-reported PA is one of the main determinants of physical frailty, a highly prevalent syndrome in MHD patients [52], accelerometers may offer a more objective characterisation of PA levels to more accurately track PA changes that could be indicative of frailty over time. In other words, accelerometers providing a detailed classification of activity intensities (e.g. MVPA vs total PA) and behaviours (e.g. step-counts, number of sit-to-stand transfers), such as the ones used in this study, may be useful for early detection and prevention of frailty in this clinical population. The wear-time recommendations in the present study are proposed with the caveat that they are the minimum requirement only to achieve an acceptable level of reliability. Adhering to a criterion of 7 days would endow accelerometer outcomes with superior reliability. However, applying such a standard with a ≥ 10 h/day wear criterion would exclude more than half of our Actigraph and ActivPAL datasets, as observed in previous studies [53, 54], seriously compromising statistical power and sample representativeness. Wear-time recommendations made here therefore balance the need for acceptable reliability while retaining an adequate sample size and preserving external validity. These recommendations may shape future study design, by shortening monitoring protocols, and help clinicians who are engaged in PA counselling to minimise patient burden and expedite data collection required for PA monitoring. However, potential bias of such purposive sampling should first be explored.

### Limitations

Reliability coefficients for some accelerometer outcomes declined as required daily wear increased reflecting the limitations of a reduced sample size. Consequently, required wear-time for a given daily wear threshold was greater for ActivPAL compared to Actigraph, particularly on non-dialysis days when the sample size was less than the minimum of 50 recommended for reliability analysis [55]. Wear-time recommendations for some ActivPAL outcomes are therefore based on the assumption that reliability coefficients would be higher with a larger sample size as observed for Actigraph, and may warrant verification in a larger cohort. In addition, it should be acknowledged that the relatively young age of participants in our study (56 years) may reflect the exclusion of patients with higher frailty levels. For instance, those who were bedbound or with severe cognitive impairment were not eligible to participate. Therefore, the generalisability of our study findings to the general ERF population on dialysis may be affected.

## Conclusions

High wear compliance observed here indicates that routine PA surveillance via accelerometry is feasible for people receiving MHD. Dialysis days are characterized by greater physical inactivity, but across days within the same condition activity behaviours are stable. Regardless of accelerometer used, a minimum of any one dialysis and two non-dialysis days with at least 8 h/day wear should provide reliable estimates of PA and sedentary behaviour. These recommendations resolve the tension between scientific rigour and retention of an adequately representative sample size.

## Supplementary information

**Additional file 1: Table S1.** Determination of Actigraph and ActivPAL derived PA outcome differences between days within the same condition; **Table S2.** Actigraph average measure ICCs for outcome variables calculated on three dialysis days; **Table S3.** Actigraph average measure ICCs for outcome variables calculated on four non-dialysis days; **Table S4.** ActivPAL average measure ICCs for outcome variables calculated on three dialysis days; **Table S5.** ActivPAL average measure ICCs for outcome variables calculated on four non-dialysis days; **Table S6.** Computed minimum wear-time requirements for Actigraph outcomes normalised to daily wear on dialysis days; **Table S7.** Computed minimum wear-time requirements for Actigraph outcomes normalised to daily wear on non-dialysis days; **Table S8.** Computed minimum wear-time requirements for ActivPAL outcomes normalised to daily wear on dialysis days; **Table S9.** Computed minimum wear-time requirements for ActivPAL outcomes normalised to daily wear on non-dialysis days; **Table S10.** Computed minimum wear-time requirements for Actigraph outcomes (not normalised to daily wear) on dialysis days; **Table S11.** Computed minimum wear-time requirements for Actigraph outcomes (not normalised to daily wear) on non-dialysis days; **Table S12.** Computed minimum wear-time requirements for ActivPAL outcomes (not normalised to daily wear) on dialysis days; **Table S13.** Computed minimum wear-time requirements for ActivPAL outcomes (not normalised to daily wear) on non-dialysis days.

## Data Availability

The datasets used and analysed during the current study are available from the corresponding author on reasonable request.

## Abbreviations

BMI: Body mass index
CPM: Counts per minute
EE: Energy expenditure
ERF: Established renal failure
Hb: Haemoglobin
ICC: Intra-class correlation coefficient
IQR: Interquartile range
MHD: Maintenance haemodialysis
MVPA: Moderate to vigorous physical activity
PA: Physical activity
SD: Standard deviation
URR: Urea reduction ratio
