# Supplementary material for: Minimum accelerometer wear-time for reliable estimates of physical activity and sedentary behaviour of people receiving haemodialysis
Source: BMC Nephrol. 2020 Jun 16;21:230. doi: 10.1186/s12882-020-01877-8 (PMC7296937; doi:10.1186/s12882-020-01877-8)
Supplement: Supplementary file 1 — Additional file 1: Table S1. Determination of Actigraph and ActivPAL derived PA outcome differences between days within the same condition; Table S2. Actigraph average measure ICCs for outcome variables calculated on three dialysis days; Table S3. Actigraph average measure ICCs for outcome variables calculated on four non-dialysis days; Table S4. ActivPAL average measure ICCs for outcome variables calculated on three dialysis days; Table S5. ActivPAL average measure ICCs for outcome variables calculated on four non-dialysis days; Table S6. Computed minimum wear-time requirements for Actigraph outcomes normalised to daily wear on dialysis days; Table S7. Computed minimum wear-time requirements for Actigraph outcomes normalised to daily wear on non-dialysis days; Table S8. Computed minimum wear-time requirements for ActivPAL outcomes normalised to daily wear on dialysis days; Table S9. Computed minimum wear-time requirements for ActivPAL outcomes normalised to daily wear on non-dialysis days; Table S10. Computed minimum wear-time requirements for Actigraph outcomes (not normalised to daily wear) on dialysis days; Table S11. Computed minimum wear-time requirements for Actigraph outcomes (not normalised to daily wear) on non-dialysis days; Table S12. Computed minimum wear-time requirements for ActivPAL outcomes (not normalised to daily wear) on dialysis days; Table S13. Computed minimum wear-time requirements for ActivPAL outcomes (not normalised to daily wear) on non-dialysis days. [file 12882_2020_1877_MOESM1_ESM.pdf]

Additional file 1: Supplemental Material

Table of contents:

Table S1. Determination of Actigraph and ActivPAL derived PA outcome differences between days within the same condition.

Table S2. Actigraph average measure ICCs for outcome variables calculated on three dialysis days.

Table S3. Actigraph average measure ICCs for outcome variables calculated on four non-dialysis days.

Table S4. ActivPAL average measure ICCs for outcome variables calculated on three dialysis days.

Table S5. ActivPAL average measure ICCs for outcome variables calculated on four non-dialysis days.

Table S6. Computed minimum wear-time requirements for Actigraph outcomes normalised to daily wear on dialysis days.

Table S7. Computed minimum wear-time requirements for Actigraph outcomes normalised to daily wear on non-dialysis days.

Table S8. Computed minimum wear-time requirements for ActivPAL outcomes normalised to daily wear on dialysis days.

Table S9. Computed minimum wear-time requirements for ActivPAL outcomes normalised to daily wear on non-dialysis days.

Table S10. Computed minimum wear-time requirements for Actigraph outcomes (not normalised to daily wear) on dialysis days.

Table S11. Computed minimum wear-time requirements for Actigraph outcomes (not normalised to daily wear) on non-dialysis days.

Table S12. Computed minimum wear-time requirements for ActivPAL outcomes (not normalised to daily wear) on dialysis days.

Table S13. Computed minimum wear-time requirements for ActivPAL outcomes (not normalised to daily wear) on non-dialysis days.

Table S1. Determination of Actigraph and ActivPAL derived PA outcome differences between days within the same condition.

Actigraph	Dialysis Days only	Non-Dialysis Days only
Wear (min)	0.93, $F_{(2,124)}$ = 2.38 p = 0.10	0.94, $F_{(3,51)}$ = 1.01 p = 0.40
Sedentary (%)	$X^{2(2)}$ = 0.13 p = 0.94	0.95, $F_{(3,51)}$ = 0.86 p = 0.47
Total PA (%)	$X^{2(2)}$ = 0.13 p = 0.94	0.95, $F_{(3,51)}$ = 0.86 p = 0.47
MVPA (%)	$X^{2(2)}$ = 0.98 p = 0.61	$X^{2(2)}$ = 4.29 p = 0.23
Steps/min	$X^{2(2)}$ = 0.60 p = 0.74	$X^{2(3)}$=1.31 p = 0.73
Triaxial cpm	$X^{2(2)}$ = 2.32 p = 0.31	$X^{2(3)}$= 3.27 p = 0.35
**ActivPAL**		
Wear (min)	0.99,$F_{(2,108)}$ = 0.21 p = 0.10	0.98,$F_{(3,44)}$ = 0.36 p = 0.79
Sit/Lie (%)	0.98,$F_{(2,108)}$ =0.51 p = 0.09	$X^{2(2)}$ = 6.65 p = 0.08
Stand (%)	0.98, $F_{(2,108)}$ =0.52 p = 0.60	$X^{2(2)}$ = 6.65 p = 0.08
Steps/min	$X^{2(2)}$ = 1.782 p = 0.41	$X^{2(3)}$= 0.932 p = 0.82
Transitions/hr	0.23, $F_{(1.7,92)}$ = 0.35 p = 0.67	$X^{2(3)}$= 5.221 p = 0.16
EE/min	$X^{2(2)}$ = 2.218 p = 0.33	$X^{2(3)}$ = 2.719 p = 0.44

MVPA = moderate to vigorous physical activity, EE = energy expenditure, cpm = counts per minute

Table S2. Actigraph average measure ICCs for outcome variables calculated on three dialysis days.

**Hours of wear**	**>4**	**>5**	**>6**	**>7**	**>8**	**>9**	**>10**
**Cases (n=)**	63	63	62	60	57	55	53
**Sedentary min/day**	0.66	0.66	0.71	0.77	0.84	0.83	0.81
**Sedentary %**	0.91	0.91	0.91	0.92	0.92	0.92	0.93
**Total PA min/day**	0.90	0.90	0.91	0.91	0.92	0.92	0.92
**Total PA %**	0.91	0.91	0.91	0.92	0.92	0.92	0.93
**MVPA min/day**	0.92	0.92	0.93	0.92	0.92	0.92	0.92
**MVPA %**	0.91	0.91	0.91	0.91	0.91	0.91	0.91
**Triax counts/day**	0.92	0.92	0.93	0.93	0.93	0.93	0.93
**Triax counts/min**	0.93	0.93	0.93	0.94	0.94	0.94	0.94
**Steps/day**	0.95	0.95	0.95	0.95	0.95	0.95	0.95
**Steps/min**	0.95	0.95	0.95	0.95	0.95	0.95	0.95

Table S3. Actigraph average measure ICCs for outcome variables calculated on four non-dialysis days.

Wear time	>4	>5	>6	>7	>8	>9	>10
Cases (n =)	54	52	52	51	40	38	32
Sedentary min/day	0.83	0.81	0.81	0.82	0.85	0.85	0.84
Sedentary %	0.92	0.93	0.93	0.93	0.92	0.92	0.87
Total PA min/day	0.92	0.93	0.93	0.93	0.92	0.92	0.90
Total PA %	0.92	0.93	0.93	0.93	0.92	0.92	0.87
MVPA min/day	0.95	0.95	0.95	0.95	0.95	0.94	0.94
MVPA %	0.95	0.95	0.95	0.95	0.95	0.94	0.94
Triax counts/day	0.94	0.94	0.94	0.94	0.93	0.93	0.93
Triax counts/min	0.95	0.95	0.95	0.95	0.95	0.95	0.94
Steps/day	0.95	0.96	0.96	0.96	0.95	0.95	0.95
Steps/min	0.96	0.96	0.96	0.96	0.96	0.96	0.95

Table S4. ActivPAL average measure ICCs for outcome variables calculated on three dialysis days.

Hours of wear	>4	>5	>6	>7	>8	>9	>10
Cases (n =)	55	55	52	51	51	49	44
Sit/Lie min/day	0.72	0.72	0.80	0.81	0.81	0.82	0.87
Sit/Lie %	0.94	0.94	0.94	0.94	0.94	0.94	0.94
Stand min/day	0.87	0.87	0.87	0.88	0.88	0.85	0.87
Stand %	0.94	0.94	0.94	0.94	0.94	0.94	0.94
Steps/day	0.90	0.90	0.92	0.92	0.92	0.91	0.91
Steps/min	0.91	0.91	0.91	0.91	0.91	0.91	0.91
Transitions/day	0.76	0.76	0.82	0.80	0.80	0.76	0.80
Transitions/hr	0.76	0.76	0.79	0.78	0.78	0.77	0.83
EE/day	0.78	0.78	0.87	0.88	0.88	0.87	0.92
EE/min	0.93	0.93	0.94	0.94	0.94	0.93	0.95

Table S5. ActivPAL average measure ICCs for outcome variables calculated on four non-dialysis days.

Hours of wear	>4	>5	>6	>7	>8	>9	>10
Cases (n =)	47	45	43	43	37	34	24
Sit/Lie mins	0.84	0.82	0.84	0.84	0.80	0.81	0.77
Sit/Lie %	0.90	0.90	0.89	0.89	0.88	0.86	0.89
Stand mins	0.89	0.90	0.88	0.88	0.88	0.85	0.90
Stand %	0.90	0.90	0.89	0.89	0.88	0.86	0.89
Steps/day	0.92	0.93	0.93	0.93	0.92	0.91	0.92
Steps/min	0.93	0.93	0.93	0.93	0.92	0.92	0.91
Transitions/day	0.82	0.83	0.83	0.83	0.82	0.76	0.73
Transitions/hr	0.88	0.89	0.89	0.89	0.89	0.87	0.84
EE/day	0.70	0.79	0.81	0.81	0.81	0.75	0.83
EE/min	0.93	0.94	0.94	0.94	0.92	0.92	0.91

Table S6. Computed minimum wear-time requirements for Actigraph outcomes normalised to daily wear on dialysis days.

	Sedentary %			Total PA %			MVPA%			Steps/min			Triaxial counts/min			Sample size for S-B computation
**Minimum wear/day**	**Wear days required for reliability level of:**			**Wear days required for reliability level of:**			**Wear days required for reliability level of:**			**Wear days required for reliability level of:**			**Wear days required for reliability level of:**			
	**0.7**	**0.8**	**0.9**	**0.7**	**0.8**	**0.9**	**0.7**	**0.8**	**0.9**	**0.7**	**0.8**	**0.9**	**0.7**	**0.8**	**0.9**	
6h	0.67	1.15	2.58	0.67	1.15	2.58	0.66	1.12	2.53	0.38	0.64	1.45	0.52	0.89	2.01	62
7h	0.58	0.99	2.24	0.58	0.99	2.24	0.67	1.16	2.60	0.36	0.61	1.38	0.47	0.81	1.82	60
8h	0.57	0.98	2.21	0.57	0.98	2.21	0.66	1.12	2.53	0.35	0.60	1.35	0.47	0.80	1.80	57
9h	0.58	1.00	2.24	0.58	1.00	2.24	0.66	1.14	2.56	0.35	0.60	1.35	0.46	0.80	1.79	55
10h	0.56	0.96	2.16	0.56	0.96	2.16	0.66	1.14	2.56	0.35	0.59	1.33	0.45	0.77	1.73	53

Table S7. Computed minimum wear-time requirements for Actigraph outcomes normalised to daily wear on non-dialysis days.

	Sedentary time %			Total PA %			MVPA%			Steps/min			Triaxial counts/min			Sample size for S-B computation
**Minimum wear/day**	**Wear days required for reliability level of:**			**Wear days required for reliability level of:**			**Wear days required for reliability level of:**			**Wear days required for reliability level of:**			**Wear days required for reliability level of:**			
	**0.7**	**0.8**	**0.9**	**0.7**	**0.8**	**0.9**	**0.7**	**0.8**	**0.9**	**0.7**	**0.8**	**0.9**	**0.7**	**0.8**	**0.9**	
6h	0.70	1.21	2.72	0.70	1.21	2.72	0.54	0.93	2.09	0.41	0.71	1.59	0.45	0.77	1.74	52
7h	0.68	1.16	2.62	0.68	1.16	2.62	0.51	0.87	1.95	0.41	0.71	1.59	0.44	0.76	1.70	51
8h	0.79	1.35	3.05	0.79	1.35	3.05	0.52	0.89	2.00	0.50	0.85	1.91	0.51	0.87	1.97	40
9h	0.80	1.38	3.10	0.80	1.38	3.10	0.55	0.95	2.13	0.49	0.84	1.89	0.51	0.87	1.97	38
10h	1.15	1.97	4.43	1.15	1.97	4.43	0.56	0.97	2.18	0.51	0.87	1.97	0.58	0.99	2.23	32

Table S8. Computed minimum wear-time requirements for ActivPAL outcomes normalised to daily wear on dialysis days.

	Sit/lie time %			Stand time %			Transfers/hour			Steps/min			Energy MET/min			Sample size for S-B computation
**Minimum wear/day**	**Wear days required for reliability level of:**			**Wear days required for reliability level of:**			**Wear days required for reliability level of:**			**Wear days required for reliability level of:**			**Wear days required for reliability level of:**			
	**0.7**	**0.8**	**0.9**	**0.7**	**0.8**	**0.9**	**0.7**	**0.8**	**0.9**	**0.7**	**0.8**	**0.9**	**0.7**	**0.8**	**0.9**	
6h	0.47	0.80	1.80	0.47	0.80	1.80	1.61	2.76	6.20	0.67	1.15	2.59	0.49	0.83	1.88	52
7h	0.46	0.79	1.77	0.46	0.79	1.77	1.69	2.90	6.52	0.65	1.12	2.52	0.48	0.82	1.84	51
8h	0.46	0.79	1.77	0.46	0.79	1.77	1.69	2.90	6.52	0.65	1.12	2.52	0.48	0.82	1.84	51
9h	0.48	0.83	1.86	0.48	0.83	1.86	1.77	3.03	6.82	0.73	1.25	2.81	0.50	0.85	1.92	49
10h	0.42	0.72	1.62	0.42	0.72	1.62	1.68	2.88	6.49	0.71	1.21	2.72	0.41	0.70	1.57	44

Table S9. Computed minimum wear-time requirements for ActivPAL outcomes normalised to daily wear on non-dialysis days.

	Sit/lie time %			Stand time %			Transfers/hour			Steps/min			Energy MET/min			Sample size for S-B computation
**Minimum wear/day**	**Wear days required for reliability level of:**			**Wear days required for reliability level of:**			**Wear days required for reliability level of:**			**Wear days required for reliability level of:**			**Wear days required for reliability level of:**			
	**0.7**	**0.8**	**0.9**	**0.7**	**0.8**	**0.9**	**0.7**	**0.8**	**0.9**	**0.7**	**0.8**	**0.9**	**0.7**	**0.8**	**0.9**	
6h	1.12	1.93	4.34	1.12	1.93	4.34	1.20	2.06	4.63	0.70	1.19	2.68	0.49	0.83	1.88	52
7h	1.12	1.93	4.34	1.12	1.93	4.34	1.20	2.06	4.63	0.70	1.19	2.68	0.48	0.82	1.84	51
8h	1.33	2.27	5.12	1.33	2.27	5.12	1.18	2.03	4.57	0.79	1.35	3.05	0.48	0.82	1.84	51
9h	1.48	2.54	5.72	1.48	2.54	5.72	1.44	2.46	5.54	0.83	1.41	3.18	0.50	0.85	1.92	49
10h	1.16	1.99	4.48	1.16	1.99	4.48	1.83	3.14	7.06	0.87	1.50	3.37	0.41	0.70	1.57	44

Table S10. Computed minimum wear-time requirements for Actigraph outcomes (not normalised to daily wear) on dialysis days.

	Sedentary mins/day			Total PA mins/day			MVPA mins/day			Steps/day			Triaxial counts/day			Sample size for S-B computation
**Minimum wear/day**	**Wear days required for reliability level of:**			**Wear days required for reliability level of:**			**Wear days required for reliability level of:**			**Wear days required for reliability level of:**			**Wear days required for reliability level of:**			
	**0.7**	**0.8**	**0.9**	**0.7**	**0.8**	**0.9**	**0.7**	**0.8**	**0.9**	**0.7**	**0.8**	**0.9**	**0.7**	**0.8**	**0.9**	
6h	2.83	4.85	10.92	0.67	1.15	2.60	0.57	0.97	2.19	0.34	0.59	1.32	0.55	0.94	2.11	62
7h	2.14	3.66	8.24	0.66	1.12	2.53	0.58	1.00	2.25	0.34	0.58	1.31	0.53	0.91	2.05	60
8h	1.33	2.29	5.14	0.64	1.09	2.45	0.60	1.02	2.30	0.35	0.61	1.36	0.52	0.89	2.00	57
9h	1.47	2.51	5.66	0.64	1.10	2.47	0.61	1.05	2.35	0.35	0.61	1.37	0.51	0.88	1.98	55
10h	1.61	2.76	6.22	0.65	1.11	2.50	0.62	1.06	2.38	0.36	0.61	1.38	0.52	0.89	2.00	53

Table S11. Computed minimum wear-time requirements for Actigraph outcomes (not normalised to daily wear) on non-dialysis days.

	Sedentary mins/day			Total PA mins/day			MVPA mins/day			Steps/day			Triaxial counts/day			Sample size for S-B computation
**Minimum wear/day**	**Wear days required for reliability level of:**			**Wear days required for reliability level of:**			**Wear days required for reliability level of:**			**Wear days required for reliability level of:**			**Wear days required for reliability level of:**			
	**0.7**	**0.8**	**0.9**	**0.7**	**0.8**	**0.9**	**0.7**	**0.8**	**0.9**	**0.7**	**0.8**	**0.9**	**0.7**	**0.8**	**0.9**	
6h	2.12	3.63	8.17	0.71	1.22	2.75	0.54	0.93	2.08	0.38	0.65	1.45	0.54	0.93	2.10	52
7h	2.04	3.49	7.86	0.70	1.19	2.69	0.51	0.87	1.97	0.34	0.59	1.32	0.54	0.93	2.10	51
8h	1.59	2.72	6.12	0.83	1.43	3.21	0.53	0.90	2.03	0.34	0.58	1.31	0.68	1.17	2.63	40
9h	1.61	2.75	6.20	0.86	1.48	3.33	0.58	0.99	2.23	0.35	0.61	1.36	0.71	1.22	2.74	38
10h	1.75	3.01	6.76	1.00	1.71	3.84	0.57	0.97	2.18	0.35	0.61	1.37	0.73	1.26	2.83	32

Table S12. Computed minimum wear-time requirements for ActivPAL outcomes (not normalised to daily wear) on dialysis days.

	Sit/lie time mins/day			Stand time mins/day			Transfers/day			Steps/day			Energy METmin/day			Sample size for S-B computation
**Minimum wear/day**	**Wear days required for reliability level of:**			**Wear days required for reliability level of:**			**Wear days required for reliability level of:**			**Wear days required for reliability level of:**			**Wear days required for reliability level of:**			
	**0.7**	**0.8**	**0.9**	**0.7**	**0.8**	**0.9**	**0.7**	**0.8**	**0.9**	**0.7**	**0.8**	**0.9**	**0.7**	**0.8**	**0.9**	
6h	1.80	3.08	6.93	1.04	1.78	4.01	1.67	2.87	6.45	0.63	1.08	2.42	1.07	1.84	4.14	52
7h	1.62	2.77	6.23	1.00	1.71	3.86	1.80	3.09	6.95	0.59	1.02	2.29	0.95	1.62	3.65	51
8h	1.62	2.77	6.23	1.00	1.71	3.86	1.80	3.09	6.95	0.59	1.02	2.29	0.95	1.62	3.65	51
9h	1.59	2.72	6.11	1.19	2.04	4.59	2.03	3.48	7.82	0.69	1.17	2.64	1.04	1.78	4.00	49
10h	1.05	1.80	4.05	1.09	1.86	4.19	1.55	2.67	6.00	0.66	1.13	2.54	0.63	1.08	2.43	44

Table S13. Computed minimum wear time requirements for ActivPAL outcomes (not normalised to daily wear) on non-dialysis days.

	Sit/lie time mins/day			Stand time mins/day			Transfers/day			Steps/day			Energy METmin/day			Sample size for S-B computation
**Minimum wear/day**	**Wear days required for reliability level of:**			**Wear days required for reliability level of:**			**Wear days required for reliability level of:**			**Wear days required for reliability level of:**			**Wear days required for reliability level of:**			
	**0.7**	**0.8**	**0.9**	**0.7**	**0.8**	**0.9**	**0.7**	**0.8**	**0.9**	**0.7**	**0.8**	**0.9**	**0.7**	**0.8**	**0.9**	
6h	1.82	3.12	7.03	1.22	2.08	4.69	1.97	3.38	7.60	0.74	1.26	2.84	2.15	3.69	8.31	52
7h	1.82	3.12	7.03	1.22	2.08	4.69	1.97	3.38	7.60	0.74	1.26	2.84	2.15	3.69	8.31	51
8h	2.38	4.07	9.16	1.26	2.16	4.86	1.99	3.41	7.67	0.81	1.39	3.13	2.24	3.85	8.66	51
9h	2.21	3.79	8.52	1.65	2.84	6.38	2.94	5.05	11.36	0.93	1.59	3.57	3.17	5.44	12.24	49
10h	2.81	4.82	10.84	1.04	1.78	4.01	3.41	5.85	13.17	0.85	1.46	3.28	1.96	3.36	7.55	44
